# Percutaneous Transluminal Angioplasty for Atherosclerotic Stenosis of Vertebral Artery Origin

**DOI:** 10.3390/jcm13144010

**Published:** 2024-07-09

**Authors:** Dagmar Krajíčková, Antonín Krajina, Roman Herzig, Vendelín Chovanec, Miroslav Lojík, Jan Raupach, Ondřej Renc, Oldřich Vyšata, Libor Šimůnek

**Affiliations:** 1 Department of Neurology, Faculty of Medicine in Hradec Králové, Charles University, 500 03 Hradec Králové, Czech Republic; dagmar.krajickova@fnhk.cz (D.K.);; 2 Department of Neurology, Comprehensive Stroke Center, University Hospital Hradec Králové, 500 05 Hradec Králové, Czech Republic; 3 Department of Radiology, Faculty of Medicine in Hradec Králové, Charles University, 500 03 Hradec Králové, Czech Republic; 4 Department of Radiology, Comprehensive Stroke Center, University Hospital Hradec Králové, 500 05 Hradec Králové, Czech Republic; 5 Research Institute for Biomedical Science, 500 02 Hradec Králové, Czech Republic

**Keywords:** vertebral artery origin, atherosclerosis, polystenotic lesions, percutaneous transluminal angioplasty, recanalization, periprocedural complications

## Abstract

**Background**: In patients with vertebral artery origin (VAO) stenosis and concomitant stenoses of other cerebral feeding arteries, data on the risk of percutaneous transluminal angioplasty (PTA) alone and with stent placement (PTAS) for VAO stenosis are limited. We aimed to determine how the presence of polystenotic lesions in other cerebral feeding arteries and concomitant carotid artery stenting (CAS) affect the periprocedural risk and long-term effect of PTA/S for atherosclerotic VAO stenosis. **Methods**: In a retrospective descriptive study, consecutive patients treated with PTA/S for ≥70% VAO stenosis were divided into groups with isolated VAO stenosis and multiple stenoses. We investigated the rate of periprocedural complications in the first 72 h and the risk of restenosis and ischemic stroke (IS)/transient ischemic attack (TIA) during the follow-up period. **Results**: In a set of 66 patients aged 66.1 ± 9.1 years, polystenotic lesions were present in 56 (84.8%) patients. 21 (31.8%) patients underwent endovascular treatment for stenosis of one or more other arteries in addition to VAO stenosis (15 underwent CAS). During the periprocedural period, no patient suffered from an IS or died, and, in the polystenotic group with concomitant CAS, there was one case of TIA (1.6%). During a mean follow-up period of 36 months, we identified 8 cases (16.3%) of ≥50% asymptomatic VA restenosis, and, in the polystenotic group, 4 (8.9%) cases of IS. **Conclusion**: The presence of severe polystenotic lesions or concomitant CAS had no adverse effect on the overall low periprocedural risk of PTA/S of VAO stenosis or the risk of restenosis during the follow-up period.

## 1. Introduction

The history of endovascular treatment of stenosis of the vertebral artery (VA) origin began in the 1980s [1,2]. Subsequently, sufficient data from observational studies and systematic reviews have been collected to confirm the high technical success rate, low periprocedural risk of the procedure [3,4,5,6,7,8], and higher risk of restenosis [5,6,7,8,9,10,11,12]. Data on the frequency of concomitant involvement of other cerebral feeding arteries and the risk of percutaneous transluminal angioplasty (PTA) both alone and with stent placement (PTAS) for stenosis of VA origin in this setting are very limited [3,13].

We aimed to determine how the presence of polystenotic lesions in other cerebral feeding arteries and concomitant carotid artery stenting (CAS) affect the periprocedural risk of PTA/S of atherosclerotic stenosis of VA origin and the long-term effect of the procedure. We hypothesized that this procedure was safe even in patients with severe polystenotic lesions.

## 2. Materials and Methods

### 2.1. Study Population

In a retrospective descriptive study, 73 consecutive PTA/S procedures in 66 patients for ≥70% atherosclerotic stenosis of VA origin since 2008 to 2019 were analyzed.

### 2.2. Recanalization Treatment

The endovascular procedure was performed under local anesthesia using a standard catheterization approach from the femoral artery via a 6F sheath (Avanti, Cordis, Miami Lakes, FL, USA). Depending on the anatomy, the subclavian artery was catheterized using a 5F diagnostic catheter or directly using a 6F guiding catheter (Guider Softtip XF, Boston Scientific, Galway, Ireland). Under road-map navigation, a 0.014 guidewire (Stabilizer Plus, Cordis, Miami Lakes, FL, USA or Transcent 300 floppy, Boston Scientific, Galway, Ireland) was introduced well into the VA through the stenosis. Sometimes oblique or cranially tilted views were necessary to visualize the stenosis. After that, a balloon expandable stent (PRO-Kinetic Energy, Biotronic, Bülach, Switzerland) was placed primarily in a large majority of cases, and a Coroflex stent (Cordis, Miami Lakes, FL, USA) was used in the remaining ten cases.

All patients were taking the best medical treatment, i.e., antiplatelet drug and statin, and possibly other drugs for risk factors for atherosclerosis, most commonly arterial hypertension, and diabetes. Three days before the procedure, all patients were loaded with dual antiplatelet therapy (100 mg of acetylsalicylic acid and 75 mg of clopidogrel daily), and full heparinization with unfractionated heparin was administered during the procedure. Patients were anticoagulated with low-molecular-weight heparin for two days after the procedure. The femoral puncture was closed with a vessel closure device (Angio-Seal Evolution 6F, Terumo Europe, Leuven, Belgium).

### 2.3. Observed Parameters

The following baseline parameters were observed: patient age and sex, presence of vascular risk factors (arterial hypertension, diabetes mellitus, hyperlipidemia, previous stroke or transient ischemic attack (TIA), ischemic heart disease, atrial fibrillation, peripheral arterial disease), concurrent impairment of other cerebral feeding arteries, history of previous carotid intervention (CAS or carotid endarterectomy (CEA)).

Patients were divided into two groups according to the presence or absence of severe stenotic/occlusive impairment of other cerebral feeding arteries. Stenosis of the VA origin was assessed as symptomatic if an ischemic lesion was confirmed in the posterior circulation using neuroimaging of the brain (computed tomography (CT) or magnetic resonance imaging (MRI)), or, in the absence of such a lesion, if clinical signs of ischemia in the posterior inferior cerebellar artery (PICA) territory were present. The causal association of isolated vertigo with the stenosis of VA origin was assessed as uncertain. Unilateral asymptomatic VA stenosis was indicated for treatment only if it was part of a polystenotic lesion.

We also investigated the technical results of vertebral angioplasty (presence of ≥30% residual stenosis) and the incidence of periprocedural complications in the first 72 h after the procedure. In the follow-up period, patients were regularly followed up at 6-month intervals clinically by neurologists (to detect new manifestations of cerebral ischemia) and by ultrasound (to detect ≥50% VA restenosis) [14].

### 2.4. Statistical Analysis

All statistical tests were performed using R version 3.5.2 (The R Foundation for Statistical Computing, Vienna, Austria). Fisher’s exact test was used for statistical analysis of categorical variables. Analyses were performed at a significance level of *p* < 0.05.

## 3. Results

**Figure 1:**
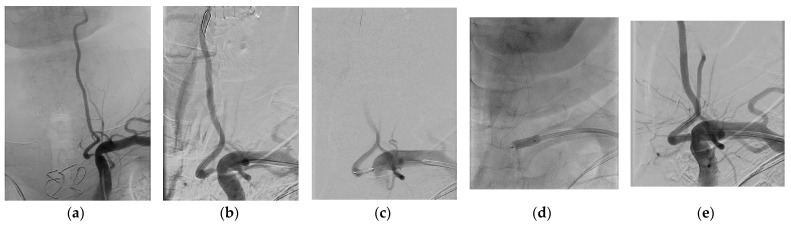
75-year-old male five days after ischemic stroke–digital subtraction angiography. (**a**) 75% ostial stenosis of the left VA, the right VA is occluded. (**b**) Using the left brachial artery approach, a 0.014-inch guidewire was inserted deep enough into the left VA. (**c**) Subsequently, a primary balloon-expandable 4 × 9 mm stent was inserted into the stenosis site. (**d**) By filling the balloon to maximum pressure, a stent was inserted. (**e**) Final angiography demonstrates full dilatation of the original stenosis. VA–vertebral artery.

From June 2008 to May 2019, we performed 73 balloon angioplasties (64 PTAS and 9 PTA) in 66 consecutive patients with ≥70% atherosclerotic stenosis of VA origin. The set consisted of 53 (80.3%) males with 59 angioplasties (80.8%) and 13 (19.7%) females with 14 (19.2%) angioplasties. A typical case is presented in Figure 1. The mean age of the cohort was 66.1 ± 9.1 years.

Severe polystenotic lesions of cerebral feeding arteries were found in 56 patients (84.8%). Contralateral VA insufficiency (occlusion/≥50% stenosis/hypo-/aplasia terminating as PICA, i.e., V4 segment aplasia) was detected in 41 patients (62.1%), and 47 patients (71.2%) had impairment (occlusion/≥70% stenosis) of the internal carotid artery (ICA). In addition, eight patients (12.1%) had a history of previous CEA procedures, and six patients (9.1%) had a history of seven CAS procedures due to severe ICA stenosis (Table 1).

Twenty-one patients (31.8%) underwent endovascular treatment of one or more cerebral feeding arteries simultaneously with PTA/S of unilateral occlusion of VA origin, and 15 of these patients (22.7% of all patients) underwent 16 CAS procedures (Table 2).

Out of the 73 angioplasties of VA origin, stenosis was symptomatic in 22 cases (30.1%), of which 20 cases had radiological evidence of a corresponding ischemic lesion on CT/MRI; two patients with negative MRI had typical clinical manifestations of ischemia in the PICA territory. The mean time from index event to PTA/S was 31.5 days. In 21 cases, the stenosis was clinically asymptomatic; in 30 cases, we assessed the association with subjective nonspecific complaints, most commonly balance disturbances, as uncertain. Severe impairment of the contralateral VA was present in 15 patients with symptomatic stenosis, in 10 patients with asymptomatic stenosis, and in 16 cases with the uncertain relationship (Table 3).

Residual ≥30% stenosis was found in 6 (8.2%) cases of the whole set, in 3 cases (4.7%) after PTAS, and in 3 cases (30%) after PTA. In the first 72 h after the procedure, none of the patients suffered from an ischemic stroke in the territory of the respective VA treated and none of them died. In a cohort of 63 angioplasties in 56 subjects with polystenotic lesions, we observed one TIA (1.6%) in the territory of the left ICA with chronic occlusion in a patient who underwent simultaneous PTAS of the right VA and right-sided CAS. In a group of 10 subjects with isolated unilateral VA impairment, one patient suffered from an amential-delirious state lasting 10 h immediately after PTAS of the right VA on day 11 after thalamic infarction.

A total of 45 (68.1%) patients with 49 (67.1%) angioplasties of VA were followed for at least 6 months. The median follow-up was 36 months. Between 7 and 126 months after PTA/S, there were 8 cases of ≥50% VA restenosis, 7 of which were in the polystenotic group. All cases of restenosis were clinically asymptomatic at the time of detection and remained asymptomatic even during the follow-up period (4 to 61 months). In 4 cases, the PTA/S procedure was repeated due to the polystenotic lesions. During the follow-up period, 4 patients suffered from ischemic stroke, all in the polystenotic group. Two ischemic strokes were located in the anterior cerebral circulation, one in the cerebellar hemisphere due to thrombotic occlusion of the basilar artery (BA) at the atherosclerotic stenosis 54 months after PTAS of VA, and one in the posterior cerebral artery (PCA) territory 36 months after PTAS of VA. No patient with ischemic stroke had evidence of VA restenosis (Table 4).

## 4. Discussion

According to our knowledge, this is the first study aiming to assess the influence of polystenotic lesions of cerebral feeding arteries on periprocedural risk and long-term effect of PTA/S in atherosclerotic stenoses of VA origin.

Concurrent with proximal VA stenosis, we performed endovascular treatment of one or more cerebral feeding arteries in 32% of patients (CAS in 23% of cases). In the group of 63 angioplasties in 56 patients with polystenotic lesions, we observed a single (1.6%) periprocedural complication, which was TIA in the territory of chronically occluded left ICA in a patient with concomitant PTAS of the right VA and with right-sided CAS. The only other complication in the entire set, this time in the group with isolated impairment of the unilateral VA, was an amential-delirious state lasting 10 h immediately after PTAS of the right VA on day 11 after thalamic infarction. Given the absence of both clinical and CT signs of a focal cerebral lesion, we did not consider this condition as an ischemic complication and concluded it as a toxoallergic reaction after contrast agent administration. In the whole set of patients, we did not observe any ischemic stroke or death, only TIA accounted for 1.4% (1/73) of periprocedural complications. The impact of polystenotic lesions and concomitant CAS on periprocedural ischemic complications could not be statistically quantified because only one complication was recorded in our cohort.

Angioplasty of the proximal segment of the VA is considered a relatively safe procedure with a low periprocedural risk of ischemic stroke and death compared to angioplasty of the distal segment of the VA. In three systematic reviews that included 600–1100 PTA/S procedures for stenosis of VA origin, only a small number of periprocedural complications was recorded. In a series of 993 VA angioplasties, of which 712 (72%) were performed for stenosis of VA origin, Stayman et al. reported the occurrence of posterior ischemic stroke in 1.2% and TIA in the vertebrobasilar territory in 0.9% of patients within 30 days of the procedure [3]. No periprocedural TIA or stroke was reported in the cohort of more than 600 angioplasties; the risk was 0.3% for death, 5.5% for periprocedural neurological complications, and 0.7% for posterior circulation stroke, with a mean follow-up of 14.2 months [4]. In a cohort of 1117 angioplasties performed in 1099 patients for stenosis of VA origin, TIA occurred in 1.5% of patients and the combined rate of stroke and death was 1.1% [7]. In a multicenter observational study published in 2013, no patients experienced an immediate procedural event or death [5].

The results of a single-center study suggest that VA hypoplasia is a common abnormality (found in 15.6% of patients with suspected stroke, more commonly (66.1%) on the right side) that can cause regional hypoperfusion in the ipsilateral PICA territory (found in 42.4% of patients examined by multimodal CT) [15]. In a group of 80 patients from the New England Medical Center Posterior Circulation Registry who had stenosis of the proximal segment of one VA, contralateral VA impairment (occlusion/stenosis/hypo-/aplasia) was found in 52.5% of cases, and 27.5% of had concomitant intracranial impairment. Hemodynamic TIA occurred in 16% of patients, 92% of whom had contralateral VA impairment [13]. In the above-mentioned systematic review of 993 PTA/S procedures for VA stenosis [3], contralateral VA stenosis/occlusion was found in 56% of patients, but periprocedural risk was not reported in this group. Dinç et al. demonstrated in a cohort of 609 patients, that male sex (*p* = 0.01) and smoking (*p* = 0.008) were associated with vertebral artery hypoplasia [16], while another paper reported that patients with atherosclerotic artery stenosis have a higher incidence of arterial hypertension (74%), diabetes mellitus (33%), and hyperhomocysteinemia (27%) [17].

In our set, polystenotic lesions were even more frequent and extensive. The contralateral VA was affected in 62% of patients: 7.6% had hypo-/aplasia, 40.9% had occlusion and 13.6% had ≥50% stenosis. In addition, 21% of patients had stenosis of the unilateral ICA, 3% of both ICAs, and 47% had ≥70% ICA stenosis. Our efforts to improve cerebral perfusion in patients with polystenotic lesions resulted in a high number of preventive angioplasties. While angioplasties for convincingly symptomatic stenosis of VA origin accounted for only 30% of all angioplasties in our set, angioplasties for asymptomatic stenosis accounted for 29% of the total, and 41% of angioplasties were due to stenosis of uncertain causality (vague and nonspecific complaints, most commonly vertigo, without radiological detection of a relevant ischemic lesion). A group of 126 PTAS procedures in the proximal VA segment in the Borgess Medical Center Vertebral Artery Ostium Stenting Registry [8] showed different percentages of each indication group, with 35% of stenoses being highly likely symptomatic, 53% likely symptomatic, and only 12% highly unlikely to have a causal relationship between stenosis and symptoms. Unfortunately, the study lacks data on possible atherosclerotic impairment of other cerebral arteries, and therefore comparison with our group is not possible. No information on possible impairment in the carotid territory was provided, so we cannot speculate on the reasons for the low percentage of angioplasty to treat asymptomatic VA stenosis (18%) in patients with 56% impairment of the contralateral VA, as reported by Stayman et al. [3].

Angioplasty of the proximal VA segment is associated with a higher risk of restenosis compared with CAS, similar to that after treatment of intracranial or coronary arteries [18,19,20,21,22,23]. Al-Ali et al. see the explanation in the similar caliber of these arteries [8]. Restenosis is usually identified in average in 20–30% of patients during long-term follow-up [5,8,9,10,11,12]. Results of meta-analyses suggest that the risk of restenosis is lower with drug-eluting stents (DES) compared with bare metal stents (BMS) [3,7,24]. Eight (16.3%) of the identified cases of ≥50% VA restenosis after BMS in our cohort are consistent with the literature. Seven cases occurred in the polystenotic group (15.6% of 45 PTA/S procedures for stenoses of VA origin) and 1 case in the group with isolated VA impairment (25% of 4 PTA/S procedures for stenoses of VA origin). Statistical analysis was limited by the significant numerical imbalance between the two groups. All restenoses during the follow-up period (mean follow-up of 36 months) remained asymptomatic.

It is currently debated whether VA angioplasty is fit for purpose, i.e., whether it reduces the risk of cerebral ischemia. The 2011 recommendations did not address this issue, stating that there is little evidence from randomized trials that endovascular treatment is superior to best medical therapy [25]. The 2018 guidelines ambiguously recommend that patients with recurrent vertebrobasilar territory symptoms (despite best medical therapy) who have 50–99% extracranial vertebral artery stenosis may be considered for revascularization [26]. Endovascular stenting is an emerging therapy for symptomatic VA ostial stenosis when medical management fails [27]. More data on the risks associated with untreated stenosis are needed to answer this question reliably. To date, the most reliable data on the risk of asymptomatic VA stenosis were provided by a study published by Compter et al., including 282 patients with asymptomatic >50% stenosis of VA origin and 3435 patients without such stenosis followed for an average of 4.6 years [28]. The risk of ischemic stroke in the posterior circulation was higher in patients with stenosis of VA origin than in patients without stenosis of VA origin (hazard ratio 4.2; 95% CI 1.4 to 13.1) and further increased in patients with stenoses of both VA origin and carotid artery (hazard ratio 10.5; 95% CI 3.0 to 37.3). The absolute risk remained low (0.4% annual stroke rate). In a study focusing on the risk of recently symptomatic VA stenosis, including 359 consecutive patients who suffered from ischemic stroke or TIA in the posterior cerebral circulation within the past 30 days, the 90-day risk of stroke was 7.2% in patients without VA stenosis, 16.2% in patients with stenosis of VA origin, and even 33% in patients with intracranial VA stenosis [29]. These results suggest that already symptomatic VA stenosis is associated with a high risk of early recurrence. The VIST and VAST randomized trials were terminated prematurely, and the small number of patients enrolled did not allow the formulation of convincing conclusions regarding the reduction of recurrent vertebrobasilar stroke [6,30]. Explaining this problem is complicated by the fact that the incidence of recurrent vertebrobasilar ischemic stroke and TIA after VA angioplasty varies widely from 0.6% to 18.1% [3,4,8,12,24]. Currently, the most convincing findings are from two meta-analyses of patients with symptomatic VA stenosis. A meta-analysis of 672 patients that compared patients treated with angioplasty plus medical therapy versus medical therapy alone showed no difference in the incidence of vascular death, any stroke, and posterior cerebral ischemia at 30 days and 1 year [31]. A meta-analysis of four studies including 370 patients came to the same conclusion [32]. Currently, although VA angioplasty is widely used in clinical practice with good technical results, evidence of its superiority over medical treatment alone is still lacking [33]. In our set, 4 patients (8.9%) suffered from ischemic stroke during the follow-up period, all in the polystenotic group. Two ischemic strokes were located in the anterior cerebral circulation, one in the cerebellar hemisphere due to thrombotic occlusion of the BA, and one in the PCA territory.

Our study has several limitations. First, its retrospective design. Second, the small number of patients in the group with isolated unilateral VA impairment limited the statistical evaluation of potential differences between the two groups. Third, it is difficult to demonstrate a direct benefit of increased perfusion after VA angioplasty in the posterior circulation as it is performed in the anterior circulation on the basis of improved cognitive functions. Moreover, collateral flow through the circle of Willis, if fully developed, may compensate for poor inflow through narrowed vertebral arteries. Fourth, the inability to evaluate the impact of individual stenoses in patients undergoing CAS reduces the significance of our study.

## 5. Conclusions

Stenoses of VA origin are very often part of polystenotic impairment of the cerebral feeding arteries. The presence of a severe polystenotic process or concomitant CAS had no negative effects on the overall low periprocedural risk from PTA/S of the stenosis of VA origin or the risk of restenosis during the follow-up period. Further studies with larger numbers of patients are needed to confirm the results of our retrospective study. These studies should be prospective and also include patients with CAS alone.

## Figures and Tables

**Table 1:** Baseline characteristic of study population.

Observed Parameter	n (%)
Patients	66 (100%)
Males	53 (80.3%)
Age, years; mean ± SD (min–max)	66.5 ± 9.1 (50–87)
Vascular risk factors	
Arterial hypertension	55 (83.3%)
Diabetes mellitus	29 (43.9%)
Hyperlipidemia	41 (62.1%)
Previous stroke/transient ischemic attack	16 (24.2%)
Ischemic heart disease	21 (31.8%)
Atrial fibrillation	7 (10.6%)
Peripheral arterial disease	19 (28.8%)
Concurrent impairment of other cerebral feeding arteries	56 (84.8%)
Occlusion of the contralateral VA	27 (40.9%)
≥50% stenosis of the contralateral VA	9 (13.6%)
Hypo-/aplasia of the contralateral VA	5 (7.6%)
Unilateral ICA occlusion	14 (21.2%)
Bilateral ICA occlusion	2 (3.0%)
≥70% stenosis of the ICA	31 (47.0%)
Occlusion of the subclavian artery/brachiocephalic trunk	4 (6.1%)
Occlusion of ≥2 arteries	11 (16.7%)
Previous carotid intervention	
Carotid endarterectomy	9 in 8 patients (12.1%)
Carotid artery stenting	7 in 6 patients (10.6%)

ICA–internal carotid artery; n–number; SD–standard deviation; VA–vertebral artery.

**Table 2:** Balloon angioplasties of vertebral and other cerebral feeding arteries.

Observed Parameter	n (%)
Angioplasties of stenosis of vertebral artery origin	73 (100%)
PTAS	64 (87.7%)
PTA	9 (12.3%)
Unilateral VA origin angioplasty + simultaneous angioplasty of another cerebral feeding artery	
+ contralateral VA origin	3 (4.5%)
+ contralateral distal VA	1 (1.5%)
+ unilateral ICA	12 (18.2%)
+ bilateral ICA	1 (1.5%)
+ unilateral ICA + unilateral SA	2 (3.0%)
+ unilateral SA	1 (1.5%)
+ bilateral SA	1 (1.5%)

ICA–internal carotid artery; n–number; PTA–percutaneous transluminal angioplasty; PTAS–percutaneous transluminal angioplasty with stent placement; SA–subclavian artery; VA–vertebral artery.

**Table 3:** Indication of angioplasty of vertebral artery origin and concurrent polystenotic lesions.

Observed Parameter	Symptomatic Stenosis n (%)	Stenosis of Uncertain Causality n (%)	Asymptomatic Stenosis n (%)
Total	22/73 (30.1%)	30/73 (41.1%)	21/73 (28.8%)
Occlusion of contralateral VA	10/22 (45.5%)	9/30 (30.0%)	8/21 (38.1%)
≥50% stenosis of contralateral VA	1/22 (4.5%)	6/30 (20.0%)	2/21 (9.5%)
Hypo-/aplasia of contralateral VA	4/22 (18.2%)	1/30 (3.3%)	0
Unilateral ICA occlusion	2/22 (9.1%)	2/30 (6.7%)	10/21 (47.6%)
Bilateral ICA occlusion	0	0	2/21 (9.5%)
≥70% stenosis of the ICA	4/22 (18.2%)	13/30 (43.3%)	14/21 (66.7%)
Concurrent CAS	2/22 (9.1%)	430 (13.3%)	10/21 (47.6%)

CAS–carotid artery stenting; ICA–internal carotid artery; n–number; VA–vertebral artery.

**Table 4:** Follow-up (median 36 months).

Observed Parameter	Total	Polystenotic Lesions n (%)	Isolated Unilateral VA Impairment n (%)	*p*
Patients	45 (100%)	41 (91.1%)	4 (8.9%)	
PTA/S of VA origin	49 (100%)	45 (91.8%)	4 (8.9%)	
≥50% restenosis of VA origin	8 (16.3%)	7 (15.6%)	1 (25.0%)	1.0
Symptomatic	0	0	0	
rePTA/S	4 (50%)	4 (57.1%)	0	
Ischemic stroke	4 (8.9%)	4 (9.8%)	0	1.0
Posterior circulation	2 (4.4%)	2 (4.9%) 1 cerebellar infarction due to BA occlusion, 1 PCA infarction	0	
≥50% restenosis of VA origin	0	0	0	
Anterior circulation	2 (4.4%)	2 (4.9%)	0	

BA–basilar artery; PCA–posterior cerebral artery; PTA/S–percutaneous transluminal angioplasty without/with stent placement; rePTA/S–repeated percutaneous transluminal angioplasty without/with stent placement; VA–vertebral artery.

## Data Availability

The datasets generated during and/or analyzed during the current study are available from the corresponding author upon reasonable request.
